# Supplementary material for: Hot spot prediction in protein-protein interactions by an ensemble system
Source: BMC Syst Biol. 2018 Dec 31;12(Suppl 9):132. doi: 10.1186/s12918-018-0665-8 (PMC6311905; doi:10.1186/s12918-018-0665-8)
Supplement: Supplementary file 2 — All relevant MATLAB code. A simple Matlab implement of our predictor. (ZIP 19,742 kb) [file 12918_2018_665_MOESM2_ESM.zip › Additional file 2/Read ASA/ASA Property/SKEMPI/1DQJC.txt]

 NetSurfP - Protein Surface Accessibility and Secondary Structure Predictions

  

|  |  |  |
| --- | --- | --- |
|  |  | NetSurfP - Protein Surface Accessibility and Secondary Structure Predictions  Technical University of Denmark |

  


---

```
# For publication of results, please cite:
# A generic method for assignment of reliability scores applied to solvent accessibility predictions.
# Bent Petersen, Thomas Nordahl Petersen, Pernille Andersen, Morten Nielsen and Claus Lundegaard
# BMC Structural Biology 2009, 9:51 doi:10.1186/1472-6807-9-51
#
# Column 1: Class assignment - B for buried or E for Exposed - Threshold: 25% exposure, but not based on RSA
# Column 2: Amino acid
# Column 3: Sequence name
# Column 4: Amino acid number
# Column 5: Relative Surface Accessibility - RSA
# Column 6: Absolute Surface Accessibility
# Column 7: Z-fit score for RSA prediction
# Column 8: Probability for Alpha-Helix
# Column 9: Probability for Beta-strand
# Column 10: Probability for Coil
E K  1DQJC                  1    0.716 147.261   1.150   0.016   0.005   0.979
E V  1DQJC                  2    0.514  79.033   1.252   0.191   0.086   0.723
B F  1DQJC                  3    0.134  26.793  -0.325   0.191   0.086   0.723
E G  1DQJC                  4    0.570  44.835   1.012   0.354   0.048   0.598
E R  1DQJC                  5    0.403  92.333   1.160   0.879   0.010   0.111
B C  1DQJC                  6    0.081  11.372   0.639   0.802   0.014   0.185
E E  1DQJC                  7    0.504  87.996   1.288   0.938   0.007   0.055
B L  1DQJC                  8    0.104  19.042   0.258   0.938   0.007   0.055
B A  1DQJC                  9    0.054   5.940   1.019   0.975   0.003   0.022
E A  1DQJC                 10    0.422  46.471   1.523   0.970   0.001   0.030
E A  1DQJC                 11    0.276  30.459   1.849   0.988   0.000   0.012
B M  1DQJC                 12    0.034   6.823   0.162   0.970   0.001   0.030
E K  1DQJC                 13    0.407  83.617   1.480   0.970   0.001   0.030
E R  1DQJC                 14    0.706 161.743   1.219   0.858   0.002   0.139
E H  1DQJC                 15    0.398  72.469   0.879   0.406   0.004   0.590
E G  1DQJC                 16    0.724  57.002  -1.036   0.016   0.005   0.979
B L  1DQJC                 17    0.080  14.611  -0.282   0.016   0.005   0.979
E D  1DQJC                 18    0.631  90.855  -0.652   0.018   0.019   0.964
E N  1DQJC                 19    0.473  69.189  -0.708   0.018   0.019   0.964
B Y  1DQJC                 20    0.175  37.483  -0.703   0.018   0.019   0.964
E R  1DQJC                 21    0.562 128.721  -0.642   0.018   0.047   0.935
E G  1DQJC                 22    0.506  39.799  -1.213   0.018   0.088   0.893
B Y  1DQJC                 23    0.226  48.253   0.404   0.064   0.216   0.721
E S  1DQJC                 24    0.295  34.609   0.114   0.064   0.216   0.721
B L  1DQJC                 25    0.051   9.338   0.321   0.191   0.086   0.723
E G  1DQJC                 26    0.288  22.658  -0.265   0.268   0.043   0.689
E N  1DQJC                 27    0.314  46.043  -0.164   0.268   0.043   0.689
B W  1DQJC                 28    0.043  10.341   0.357   0.321   0.252   0.427
B V  1DQJC                 29    0.036   5.595   0.189   0.169   0.612   0.219
B C  1DQJC                 30    0.021   2.906   0.959   0.157   0.773   0.069
B A  1DQJC                 31    0.049   5.433   0.596   0.085   0.775   0.140
B A  1DQJC                 32    0.024   2.645   0.698   0.085   0.775   0.140
B K  1DQJC                 33    0.169  34.681   0.320   0.085   0.775   0.140
B F  1DQJC                 34    0.212  42.568  -0.706   0.085   0.775   0.140
B E  1DQJC                 35    0.149  26.013  -0.881   0.074   0.484   0.442
B S  1DQJC                 36    0.167  19.549  -1.399   0.066   0.296   0.638
E N  1DQJC                 37    0.433  63.464  -0.926   0.056   0.142   0.802
B F  1DQJC                 38    0.233  46.823  -0.441   0.018   0.088   0.893
E N  1DQJC                 39    0.394  57.740  -0.158   0.019   0.141   0.840
E T  1DQJC                 40    0.335  46.437  -1.379   0.020   0.205   0.775
E Q  1DQJC                 41    0.577 103.016  -0.500   0.022   0.359   0.619
E A  1DQJC                 42    0.310  34.162  -0.346   0.022   0.552   0.426
E T  1DQJC                 43    0.372  51.555  -0.004   0.023   0.655   0.322
E N  1DQJC                 44    0.333  48.766  -0.371   0.022   0.552   0.426
E R  1DQJC                 45    0.502 115.050  -0.194   0.022   0.359   0.619
E N  1DQJC                 46    0.432  63.230  -0.775   0.019   0.141   0.840
E T  1DQJC                 47    0.626  86.896  -1.138   0.018   0.047   0.935
E D  1DQJC                 48    0.478  68.880  -1.869   0.053   0.043   0.903
E G  1DQJC                 49    0.462  36.336  -1.638   0.018   0.019   0.964
E S  1DQJC                 50    0.329  38.524  -1.227   0.053   0.043   0.903
E T  1DQJC                 51    0.568  78.823  -0.529   0.053   0.043   0.903
E D  1DQJC                 52    0.377  54.254  -1.666   0.053   0.043   0.903
B Y  1DQJC                 53    0.290  62.016  -0.270   0.053   0.043   0.903
B G  1DQJC                 54    0.168  13.182  -2.160   0.113   0.087   0.800
B I  1DQJC                 55    0.100  18.556   0.538   0.125   0.227   0.648
B L  1DQJC                 56    0.093  17.028  -0.241   0.148   0.418   0.435
B Q  1DQJC                 57    0.130  23.182  -0.453   0.074   0.484   0.442
B I  1DQJC                 58    0.052   9.712  -0.761   0.074   0.484   0.442
E N  1DQJC                 59    0.296  43.290  -0.259   0.066   0.296   0.638
E S  1DQJC                 60    0.382  44.817  -1.407   0.056   0.142   0.802
E R  1DQJC                 61    0.356  81.593   0.036   0.066   0.296   0.638
E W  1DQJC                 62    0.283  68.037  -0.051   0.069   0.386   0.545
B W  1DQJC                 63    0.160  38.528   0.019   0.135   0.317   0.548
B C  1DQJC                 64    0.033   4.675  -1.633   0.118   0.150   0.732
E N  1DQJC                 65    0.425  62.249   0.250   0.113   0.087   0.800
E D  1DQJC                 66    0.543  78.318  -0.196   0.018   0.019   0.964
E G  1DQJC                 67    0.611  48.117  -1.664   0.018   0.019   0.964
E R  1DQJC                 68    0.561 128.515  -0.715   0.018   0.019   0.964
E T  1DQJC                 69    0.484  67.117  -1.081   0.018   0.019   0.964
E P  1DQJC                 70    0.517  73.376  -1.612   0.018   0.019   0.964
E G  1DQJC                 71    0.533  41.947  -1.566   0.018   0.019   0.964
E S  1DQJC                 72    0.403  47.278  -0.851   0.018   0.019   0.964
E R  1DQJC                 73    0.502 114.981  -0.180   0.053   0.043   0.903
E N  1DQJC                 74    0.399  58.414  -1.298   0.053   0.043   0.903
E L  1DQJC                 75    0.351  64.250  -0.230   0.113   0.043   0.844
B C  1DQJC                 76    0.108  15.121  -1.045   0.113   0.087   0.800
E N  1DQJC                 77    0.633  92.627   0.024   0.056   0.142   0.802
B I  1DQJC                 78    0.170  31.431   0.417   0.019   0.141   0.840
E P  1DQJC                 79    0.490  69.588   1.400   0.052   0.084   0.864
B C  1DQJC                 80    0.039   5.462  -0.142   0.257   0.016   0.727
E S  1DQJC                 81    0.623  72.980   0.871   0.339   0.016   0.645
E A  1DQJC                 82    0.554  61.018   1.173   0.406   0.004   0.590
B L  1DQJC                 83    0.141  25.799   0.598   0.321   0.003   0.675
B L  1DQJC                 84    0.239  43.688   0.347   0.176   0.004   0.820
E S  1DQJC                 85    0.463  54.205   0.675   0.053   0.005   0.942
E S  1DQJC                 86    0.648  75.946  -0.201   0.176   0.004   0.820
E D  1DQJC                 87    0.513  73.866   0.639   0.109   0.005   0.886
B I  1DQJC                 88    0.053   9.787   0.812   0.600   0.003   0.397
E T  1DQJC                 89    0.382  53.053   1.207   0.694   0.003   0.303
E A  1DQJC                 90    0.399  43.915   0.878   0.858   0.002   0.139
B S  1DQJC                 91    0.087  10.150   0.359   0.923   0.002   0.076
B V  1DQJC                 92    0.041   6.240  -0.006   0.970   0.001   0.030
E N  1DQJC                 93    0.403  58.985   1.808   0.988   0.000   0.012
B C  1DQJC                 94    0.029   4.029   0.696   0.988   0.000   0.012
B A  1DQJC                 95    0.021   2.270   0.890   0.988   0.000   0.012
E K  1DQJC                 96    0.259  53.338   1.004   0.988   0.000   0.012
E K  1DQJC                 97    0.356  73.270   1.767   0.970   0.001   0.030
B I  1DQJC                 98    0.025   4.643   0.685   0.975   0.003   0.022
B V  1DQJC                 99    0.102  15.616   0.497   0.938   0.007   0.055
E S  1DQJC                100    0.523  61.331   0.361   0.802   0.014   0.185
E D  1DQJC                101    0.426  61.401   0.442   0.502   0.002   0.495
E G  1DQJC                102    0.487  38.343  -0.607   0.246   0.004   0.750
E N  1DQJC                103    0.514  75.220  -0.579   0.109   0.005   0.886
E G  1DQJC                104    0.386  30.362  -1.000   0.058   0.017   0.925
B M  1DQJC                105    0.114  22.791   0.303   0.113   0.043   0.844
E N  1DQJC                106    0.479  70.184  -0.392   0.118   0.150   0.732
B A  1DQJC                107    0.111  12.276   0.070   0.428   0.171   0.402
B W  1DQJC                108    0.093  22.463   0.071   0.605   0.105   0.290
E V  1DQJC                109    0.467  71.824   0.411   0.660   0.049   0.291
B A  1DQJC                110    0.201  22.161  -0.828   0.660   0.049   0.291
B W  1DQJC                111    0.088  21.212   0.792   0.660   0.049   0.291
E R  1DQJC                112    0.318  72.868   1.265   0.622   0.015   0.363
E N  1DQJC                113    0.634  92.759   0.914   0.522   0.016   0.462
E R  1DQJC                114    0.404  92.608   0.967   0.430   0.016   0.555
B C  1DQJC                115    0.036   4.998  -1.796   0.257   0.016   0.727
E K  1DQJC                116    0.614 126.300   0.877   0.115   0.016   0.868
E G  1DQJC                117    0.762  59.985  -0.998   0.018   0.019   0.964
E T  1DQJC                118    0.527  73.053   1.433   0.018   0.019   0.964
E D  1DQJC                119    0.642  92.440   1.134   0.018   0.019   0.964
B V  1DQJC                120    0.066  10.129   0.553   0.246   0.004   0.750
E Q  1DQJC                121    0.568 101.516   0.402   0.321   0.003   0.675
E A  1DQJC                122    0.602  66.340   1.050   0.406   0.004   0.590
B W  1DQJC                123    0.210  50.433   0.786   0.502   0.002   0.495
B I  1DQJC                124    0.144  26.733  -0.095   0.522   0.016   0.462
E R  1DQJC                125    0.643 147.178   1.111   0.522   0.016   0.462
E G  1DQJC                126    0.605  47.653   0.810   0.257   0.016   0.727
B C  1DQJC                127    0.140  19.684  -0.252   0.053   0.043   0.903
E R  1DQJC                128    0.688 157.438   0.724   0.018   0.088   0.893
E L  1DQJC                129    0.513  93.967  -0.469   0.000   0.001   0.999

Explain the output.  Go back.
```

---
